# Students must not be collateral damage in immigration clampdowns

**DOI:** 10.1038/s44319-025-00531-4

**Published:** 2025-07-31

**Authors:** Shina Caroline Lynn Kamerlin

**Affiliations:** Georgia Institute of Technology, School of Chemistry and Biochemistry, Atlanta, GA USA

**Keywords:** Careers, Economics, Law & Politics

## Abstract

Amid escalating anti-immigrant sentiments, international students pose an easy target in “immigration wars”, with heavy human and professional cost.

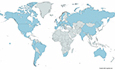

I am an adult “third culture kid”, the child of parents from two different continents and cultures, and born in a third country: a citizen of everywhere and nowhere at once. By the time I started university, I had lived in four countries on two continents, and spoke four languages fluently. I grew up in international schools and/or studying in international programs, surrounded by children and teenagers from across the world. I have kept that environment up as I transitioned to my own independent career: since starting my own laboratory in 2011, more than 120 researchers at all career stages have come to work with our team, hailing from 31 countries across the globe and every continent (Fig. 1). I firmly believe that the most productive research teams rely on and attract the best talent from every country and background, harnessing diverse ways of thinking and approaching scientific problems to tackle challenging and ground-breaking problems.

**Figure 1:**
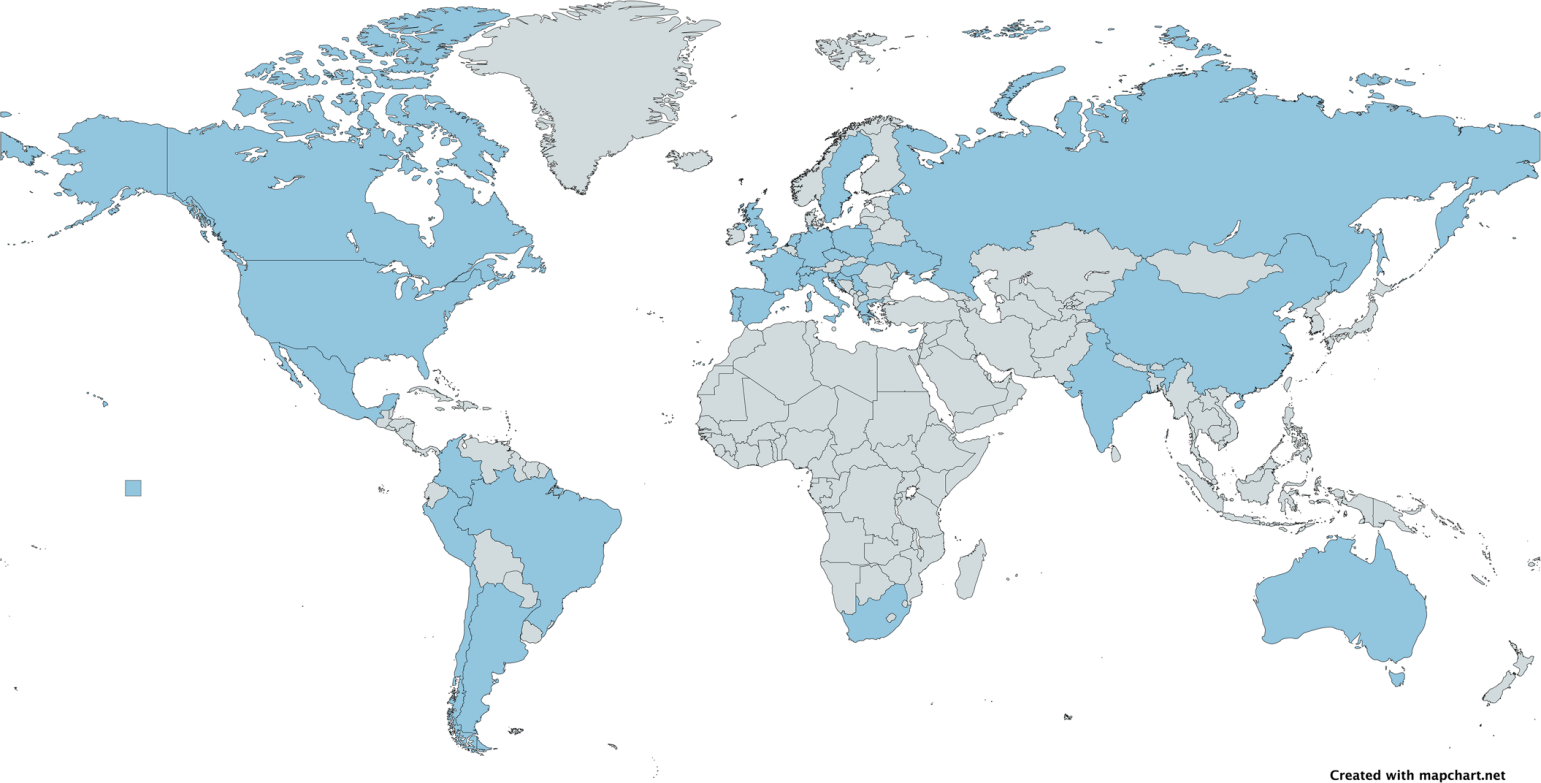
A map of the different countries (in blue) where both members of and visitors to my research team have come from over the past fourteen years. Map created using MapChart under a CC BY-SA 4.0 license.

In the context of this background, it breaks my heart to see the tide of right-wing populism both in Europe and globally, and the anti-immigrant sentiments in its wake. Further, as an academic researcher, it alarms me to see how frequently students become the hostages of politicians pandering to this right-wing anti-immigrant sentiment. The alarming situation in the USA is receiving widespread attention (Pulverer, 2025), and it is not surprising that international students are either themselves considering or being advised to consider other options, including, for example, redirecting to the UK as a fellow English-speaking country.

However, the UK is also clamping down on international students. The UK government restricted the time students can remain after graduation from 2 years to 18 months, deterring about half of international students. Further, it is proposing a levy on university income from international students, a so-called “foreign student tax”. This is on top of the fact that non-UK—including EU—students have to pay for UK healthcare as part of their immigration application. Restrictions have also been placed on the ability of international students to bring their families to the UK, unless studying for a research postgraduate degree. Meanwhile, international students are being maligned as using their visas as a ”backdoor” to stay in the UK, with claims that “UK higher education supplies an alibi for a failed migration policy”.

This anti-international-student trend is not limited to the UK and USA: Canada is set to increase the requirements for financial support for international students, and to maintain the caps on the number of international students. Australia is similarly attempting to slow down foreign student visa applications. However, the drastic reduction of options for international students also happens outside the Anglophone world. As just some examples: Norway attempted to implement—and eventually decided to scrap—mandatory Norwegian language training for foreign PhD students and postdocs; in the Netherlands the majority of undergraduate courses are taught in Dutch; Denmark is restricting work and family mobility rights for third-country students that are enrolled in non-state-approved higher educational programs; and universities in the Netherland are working to “rebalance internationalization”, at the same time as international students are being blamed for causing a housing crisis in the country, and are feeling increasingly unwelcome.

Unfortunately, therefore, although the situation in the USA is highly alarming (Pulverer, 2025), it is not only there where anti-immigration populists seeking to restrict the number of migrants see an easy target in the form of international students. This really is an example, however, of cutting off your nose to spite your face. In 2024, according to NAFSA, international students contributed a record-breaking level of spending, as well as 378,000 jobs to the US economy. In the UK, they similarly boosted the UK economy by almost £42 billion in 2023. In 2022, international students in Canada are estimated to have contributed $30.9 billion CAD (1.2%) to Canada’s GDP. Similar data can be found for other countries, where the economic impact of international students has been substantive.

Not all countries are closing their doors to international students. France and Germany are both actively working to increase their numbers, while Sweden is introducing more favorable visa terms for them. Conversely, countries that are proactively targeting international student recruitment are facing hits, most notably the UK, where the economic impact has been significant due to the over-reliance of UK institutions on international student tuition, which is substantially higher than domestic tuition. This, in turn, has been contributing to the ongoing funding crisis at UK universities, which has impacted more than 40% of institutions, and caused several universities to teeter on the brink of bankruptcy. On top of this, it has also been proposed to restrict visa applications for some nationalities, including people from Pakistan, Nigeria, and Sri Lanka.

Although student mobility is being restricted in a less dramatic fashion than currently in the USA, the impact on science will be no less harmful, as PIs are unable to recruit talent broadly, and miss out on outstanding students and researchers in their teams. By restricting work options and residency paths during and after education, host countries lose out on the talent and skills of the students they themselves have just trained. In this context, in what seems to be a U-turn on migration policy, Prime Minister Georgia Meloni of Italy plans to issue nearly 500,000 non-EU work visas over the next 3 years to strengthen the Italian workforce. Beyond the economic aspects, there are also the human aspects as students are stripped of opportunities, and face increasing uncertainty in what is already a highly volatile world. The fact that international students are an easy target of populist policies does not make this an ethical path, and it is beyond time to stop taking students hostage in immigration wars.

## Supplementary information

Peer Review File

## References

[CR1] Pulverer B (2025) Student hostages in culture war. EMBO Rep 10.1038/s44319-025-00527-0 10.1038/s44319-025-00527-0 PMC12373890 40745395

