# Supplementary material for: Students must not be collateral damage in immigration clampdowns
Source: EMBO Rep. 2025 Jul 31;26(16):3975–6. doi: 10.1038/s44319-025-00531-4 (PMC12373955; doi:10.1038/s44319-025-00531-4)
Supplement: Supplementary file 1 — Peer Review File [file 44319_2025_531_MOESM1_ESM.pdf]

EMBO reports

Students must not be collateral damage in immigration clampdowns

Shina Caroline Lynn Kamerlin

Corresponding author(s): Shina Caroline Lynn Kamerlin (skamerlin3@gatech.edu)

Review Timeline:

Submission Date:	11th Jul 25
Accepted:	11th Jul 25

Editor: Holger Breithaupt

Transaction Report: This article type is not peer reviewed.
